# Influenza- and COVID-19-Associated Pulmonary Aspergillosis: Are the Pictures Different?

**DOI:** 10.3390/jof7050388

**Published:** 2021-05-15

**Authors:** Florian Reizine, Kieran Pinceaux, Mathieu Lederlin, Brice Autier, Hélène Guegan, Arnaud Gacouin, David Luque-Paz, Christelle Boglione-Kerrien, Astrid Bacle, Brendan Le Daré, Yoann Launey, Mathieu Lesouhaitier, Benoit Painvin, Christophe Camus, Alexandre Mansour, Florence Robert-Gangneux, Sorya Belaz, Yves Le Tulzo, Jean-Marc Tadié, Adel Maamar, Jean-Pierre Gangneux

**Affiliations:** 1 CHU Rennes, Maladies Infectieuses et Réanimation Médicale, F-35033 Rennes, France; kieran.pinceaux@chu-rennes.fr (K.P.); arnaud.gacouin@chu-rennes.fr (A.G.); david.luque-paz@chu-rennes.fr (D.L.-P.); mathieu.lesouhaitier@chu-rennes.fr (M.L.); benoit.painvin@chu-rennes.fr (B.P.); christophe.camus@chu-rennes.fr (C.C.); alexandre.mansour@chu-rennes.fr (A.M.); yves.le.tulzo@chu-rennes.fr (Y.L.T.); jeanmarc.tadie@chu-rennes.fr (J.-M.T.); adel.maamar@chu-rennes.fr (A.M.); 2 CHU Rennes, Service d’Imagerie Médicale, F-35033 Rennes, France; mathieu.lederlin@chu-rennes.fr; 3 CHU Rennes, Service de Parasitologie-Mycologie, F-35033 Rennes, France; brice.autier@chu-rennes.fr (B.A.); helene.guegan@chu-rennes.fr (H.G.); florence.robert-gangneux@univ-rennes1.fr (F.R.-G.); sorya.belaz@chu-rennes.fr (S.B.); 4 Univ Rennes, CHU Rennes, Inserm, EHESP, Irset (Institut de Recherche en Santé, Environnement et Travail)—UMR_S 1085, F-35000 Rennes, France; astrid.bacle@univ-rennes1.fr; 5 CHU Rennes, Service de Pharmacologie, F-35033 Rennes, France; christelle.boglione-kerrien@chu-rennes.fr; 6 CHU Rennes, Service de Pharmacie, F-35033 Rennes, France; brendan.le.dare@chu-rennes.fr; 7 CHU Rennes, Service de Réanimation Chirurgicale, F-35033 Rennes, France; yoann.launey@chu-rennes.fr

**Keywords:** COVID-19, influenza, pulmonary aspergillosis, CAPA, IAPA, corticosteroids, acute respiratory distress syndrome, CT-scan, voriconazole, therapeutic drug monitoring

## Abstract

Invasive pulmonary aspergillosis (IPA) in intensive care unit patients is a major concern. Influenza-associated acute respiratory distress syndrome (ARDS) and severe COVID-19 patients are both at risk of developing invasive fungal diseases. We used the new international definitions of influenza-associated pulmonary aspergillosis (IAPA) and COVID-19-associated pulmonary aspergillosis (CAPA) to compare the demographic, clinical, biological, and radiological aspects of IAPA and CAPA in a monocentric retrospective study. A total of 120 patients were included, 71 with influenza and 49 with COVID-19-associated ARDS. Among them, 27 fulfilled the newly published criteria of IPA: 17/71 IAPA (23.9%) and 10/49 CAPA (20.4%). Kaplan–Meier curves showed significantly higher 90-day mortality for IPA patients overall (*p* = 0.032), whereas mortality did not differ between CAPA and IAPA patients. Radiological findings showed differences between IAPA and CAPA, with a higher proportion of features suggestive of IPA during IAPA. Lastly, a wide proportion of IPA patients had low plasma voriconazole concentrations with a higher delay to reach concentrations > 2 mg/L in CAPA vs. IAPA patients (*p* = 0.045). Severe COVID-19 and influenza patients appeared very similar in terms of prevalence of IPA and outcome. The dramatic consequences on the patients’ prognosis emphasize the need for a better awareness in these particular populations.

## 1. Introduction

Invasive pulmonary aspergillosis (IPA) has been mainly described in patients with severe neutrophil dysfunction, especially those with prolonged neutropenia [1]. Increasing evidence shows that critically ill patients are at risk of IPA [2,3,4]. Risk factors for IPA development in these critical patients are heterogeneous. Lower respiratory tract impairment, prolonged mechanical ventilation, corticosteroid administration, or immunological dysfunction are often involved [2]. The diversity of the patient backgrounds is reflected by their clinical and biological presentation and thus the criteria that should be used for case definition. Generic consensus definitions, such as those of the European Organization for Research and Treatment of Cancer/Mycosis Study Group Education and Research Consortium (EORTC/MSGERC) or the AspICU [1,3,5], are sometimes not adapted to specific groups of patients [6].

Influenza-associated pulmonary aspergillosis (IAPA) is an emerging complication of influenza infection, often associated with *Aspergillus* tracheobronchitis [7], that markedly increases influenza-associated mortality [8]. Commonly recognized elements of IAPA include epithelial damage, NADPH-oxidase impairment and the modulation of immune function directly due to the virus, although the pathogenesis is still unclear [7]. IAPA cases exhibit atypical clinical features, which influence the results of diagnostic tests, such as broncho-alveolar lavage (BAL) and serum galactomannan (GM) or respiratory sample culture, as well as atypical radiological features [7]. This led to the recent proposition of new criteria for IAPA case definition in intensive care unit (ICU) patients [7]. Unlike IPA observed in immunocompromised patients, the radiological patterns of acute respiratory distress syndrome (ARDS) patients are much more difficult to interpret and the particularities of IPA imaging in these situations have thus far been only poorly evaluated.

The background of patients suffering from Coronavirus Disease 2019 (COVID-19) also appears to be highly compatible with the occurrence of IPA [9]. ICU patients admitted for severe acute respiratory syndrome coronavirus 2 (SARS-CoV-2) and influenza virus infections share specific features, commonly described to increase the risk for ventilator associated pneumonia [10] and particularly fungal diseases including ARDS, possible corticosteroid administration, and systemic dysregulation of immune function [11]. The incidence of COVID-19-associated pulmonary aspergillosis (CAPA) in ICU patients varies according to the major national cohorts from the United Kingdom (14.1%), Italy (27.7%), Germany (26.3%), the Netherlands (19.4%), and France (National MYCOVID clinical trial: 19.6% probable and possible CAPA) [12,13,14,15,16] and are similar to the rate of IAPA observed in ICU cohorts (19%) from Belgium and the Netherlands [8]. Nevertheless, the situation appears not to be strictly transposable between the two co-infections, as, for example, *Aspergillus* tracheobronchitis is not commonly described in CAPA and serum GM is much less frequently positive in CAPA than IAPA [1,7]. However, a more accurate comparison between CAPA and IAPA has until recently been difficult due to the absence of a clear CAPA case definition, as highlighted by some authors [15].

The recently published CAPA case definition [17] has made it possible to compare patients suffering from IAPA and CAPA. This will improve our epidemiological knowledge concerning CAPA, which is still incomplete, and represents the first step towards improving the management of the COVID-19-associated fungal risk [18,19]. In this monocentric retrospective study, we aimed to compare the demographic, clinical, radiological, and biological features and outcomes of IAPA and CAPA cases in ICU applying the recently proposed case definitions of CAPA.

## 2. Methods

### 2.1. Populations

All patients who were admitted from 20 September 2009 to 8 February 2020 to the ICUs of the Rennes University Hospital for influenza-associated ARDS and underwent a mycological analysis of BAL, tracheal aspirate, or sputum were included in the study as “influenza patients’’ (*n* = 71). All patients who were admitted to the same unit for COVID-19 from 3 March to 9 September 2020 were included as “COVID-19 patients’’ (*n* = 49). The COVID-19 patients were strictly monitored for fungal infections twice weekly based on tracheal aspirates, as detailed in a previous publication [20]. Influenza infection and COVID-19 were confirmed by RT-PCR of respiratory samples or nasopharyngeal swabs using the Influenza A/B r-gene^TM^ (Argene^®^, bioMérieux, Marcy-l’Etoile, France) and TaqPath™ COVID-19 (Thermo Fisher Scientific, Illkirch-Grafenstaden, France) assays. ARDS was defined according to international guidelines [21]. Epidemiological and clinical data were collected during hospitalization. A blood count and biochemical check-up, including the measurement of creatinine levels, were performed at the beginning of the hospitalization for each patient. For statistical analysis, patients were classified following the AspICU [3], IAPA [7], and CAPA [17] criteria when specified. Data presented in tables and figures were extracted from each patient’s medical records. The simplified acute physiology score (SAPS) II was assessed within 24 h following ICU admission and the Sepsis-Related Organ Failure Assessment (SOFA) score was calculated on days 1 and 5. This study conforms to the principles outlined in the Declaration of Helsinki and was approved by the institutional ethics board of Rennes University Hospital, France (N 20-56).

### 2.2. Detection of Aspergillus in Respiratory Samples by Culture and PCR

Fungal culture was performed from respiratory samples in Sabouraud-Chloramphenicol media, inoculated with 100 µL of pellets, and incubated for seven days at 30 °C and 37 °C. *Aspergillus* isolates were identified at the genus level based on microscopic features. *Aspergillus* fungi were then identified at the species level by MALDI-ToF mass spectrometry after fungal colony extraction [22] using a MALDI Biotyper device (Bruker France, Marne-la-Vallée, France) and the Mass Spectrometry Identification (MSI) database for the identification of fungi [23].

The molecular detection of *Aspergillus* was also performed on respiratory samples after DNA extraction. Briefly, 200 µL of a BAL pellet or other liquefied respiratory samples were first incubated overnight at 56 °C with proteinase K (Qiagen France, Les Ulis, France). DNA was then extracted using the manual QIAamp DNA Mini Kit (Qiagen) or the automated EZ1 Advanced XL system (Qiagen) using the EZ1 DSP Virus Kit (Qiagen). *Aspergillus* qPCR assays were performed as previously described, targeting either an *Aspergillus* mitochondrial gene or an *Aspergillus* 28S rDNA region [24], depending on the period of inclusion.

### 2.3. Detection of Aspergillus Galactomannan (GM) in Blood and Respiratory Samples

GM measurement was performed in serum with an index cutoff > 0.5 and in BAL with an index cutoff > 1 using the Platelia GM *Aspergillus* assay (Bio-Rad, Marnes-la-Coquette, France) following the manufacturer’s recommendations. GM detection in non-bronchoscopic lavage respiratory samples was performed using the sōna *Aspergillus* lateral flow assay (LFA) (IMMY diagnostics, OK, USA), following the manufacturer’s recommendations, due to the biological hazard for laboratory workers. Quantitative results were obtained by reading the LFA with the sōna cube reader (IMMY diagnostics).

### 2.4. Imaging

Chest computed tomography (CT) scans of patients who developed IPA were analyzed by a senior radiologist who was blinded to the IAPA or CAPA status. The following items were categorized as absent or present as generally reported in the literature: diffuse reticular or alveolar opacities, wedge-shaped segmental or lobar consolidation, well-circumscribed nodules, halo signs, cavitation, air crescent signs, tree in bud, bronchial wall thickening, and pleural effusion [1,3,7].

### 2.5. Therapeutic Drug Monitoring

Among patients treated with voriconazole, therapeutic drug monitoring was performed after the initiation of this treatment. A plasmatic voriconazole trough concentration (VTC) target between 2 and 6 mg/L was recommended as a voriconazole therapeutic range in ICU IPA patients [25].

Trough concentrations (=Cmin), defined as concentrations measured 12 ± 2 h after voriconazole administration, were measured from day 3 after the beginning of the treatment, after achievement of steady-state. Blood samples were centrifuged at 3200× *g* for 10 min, and then stored at −20 °C prior to being assayed. Voriconazole plasma concentrations were determined using a validated liquid chromatography tandem mass spectrometry assay. The linearity range of the assay extends from 0.1 to 12 mg/L.

### 2.6. Statistical Analysis

Demographic and clinical characteristics of patients are presented as numbers and percentages for categorical variables and medians and interquartile ranges (IQR, 25–75%) for continuous variables. The Mann–Whitney U test was used for quantitative data and qualitative data were compared using the Chi-square or Fisher test, as appropriate. Survival curves were constructed until day 90 from the diagnosis of ARDS using the Kaplan–Meier method and were compared using the log rank test. Two-sided tests were performed and reached statistical significance when the *p*-value was <0.05.

All statistical analyses were performed using GraphPad Prism 8.4 (GraphPad Software, La Jolla, CA, USA) and R Statistical Software 3.5.2 (R Foundation for Statistical Computing, Vienna, Austria).

## 3. Results

Overall, 120 patients were included in the study, 71 admitted for severe influenza and 49 for severe COVID-19. Among them, 27 (22.5%) presented with COVID-19- (CAPA) and Influenza- (IAPA) associated pulmonary aspergillosis. Among the 10/49 (20.4%) CAPA patients, four were probable CAPA and six possible CAPA according to the most recent consensual definitions by Koehler et al. [17]. Among the 17/71 (23.9%) IAPA patients, 13 fulfilled the definitions by Verweij et al. [7] and four the definitions by Blot et al. [3]. A comparative analysis of these two groups of IAPA patients showed no significant differences in terms of background, severity, or outcome. Thus, we considered all 17 patients to constitute the same IAPA group during this study. The IAPA diagnosis was based on at least one positive GM result for the serum (*n* = 7/17, 41.1%) and/or BAL (*n* = 10/17 patients, 58.8%) and/or a fungal culture of *A. fumigatus* from respiratory samples (*n* = 15/17, 88.2%). The CAPA diagnosis was based on at least one positive GM result for serum (*n* = 3/10, 30%) and/or an *A. fumigatus*-positive culture from a non-bronchoscopic lavage (*n* = 9/10 patients, 90%) and/or combined positivity of GM and *A. fumigatus* PCR in non-bronchoscopic lavage (*n* = 1/10, 10%). Mycological arguments that allowed the IPA classification are presented for each patient in Table S1.

Demographic and admission characteristics of the patients according to their aspergillosis status are summarized in Table 1. The median age was 59 years and 80 (66%) of 120 patients were men. There were no significant differences in demographic or admission characteristics between patients with and without IPA. CAPA patients were significantly older than IAPA patients (mean ages 72 and 58 years, *p* = 0.036). The proportion of immunosuppressed patients was numerically higher among patients with IPA (37%, 10/27) than those without (20%, 19/93), but the difference did not reach statistical significance (*p* = 0.076). Similarly, the frequency of immunosuppressed patients was lower among CAPA patients (20%) than IAPA patients (47.1%) without reaching statistical difference. Among recognized risk factors for IPA, solid cancers and hematological malignancies were observed for 25.9% of IPA patients and 12.9% of patients without IPA (*p* = 0.13). A summary of reported cases is presented in Table S2. The frequency of patients with neoplasia was lower in CAPA (10%) than IAPA patients (35.3%), without reaching statistical difference.

**Figure 1:**
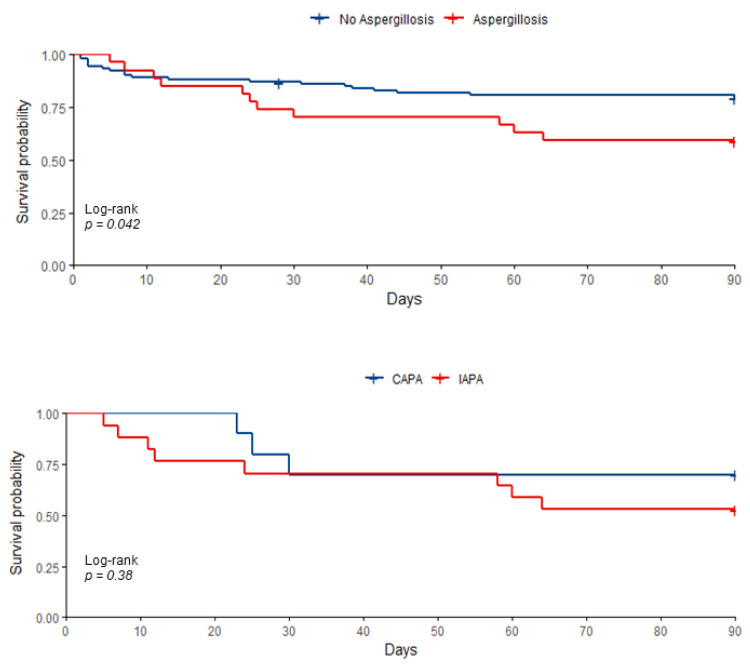
Cumulative 90-day mortality from admission to the intensive care unit in the whole population (**Top**) and among CAPA and IAPA patients (**Below**).

The biological data at ICU admission are summarized in Table 1 and show that the CAPA patients were globally less severely ill than the IAPA patients. The SOFA score on day 1 was significantly lower for CAPA than IAPA patients (*p* = 0.012). Survival analysis at day 90 showed higher mortality among all IPA patients (*p* = 0.042), whereas mortality did not differ between those with CAPA and IAPA (Figure 1).

In this cohort, the duration of mechanical ventilation was higher for patients with IPA (23 days [IQR 17–40] than those without (17 days [IQR 9–25], *p* = 0.038) (Table 1). Renal replacement therapy was more frequent for IPA patients (*p* = 0.027) and supportive therapy by extracorporeal membrane oxygenation (ECMO) was less frequent for CAPA than IAPA patients (*p* = 0.004). Patients who developed IPA were more frequently treated by corticosteroids (70.4% vs. 38.7%, *p* = 0.004) and the frequency of such treatment was similar for the CAPA and IAPA groups. The median ICU length of stay was longer for IPA patients (25 days [IQR 19–48] vs. 19 days [IQR 12–30], *p* = 0.04). Finally, survival at 90 days after ICU admission was 59.3% for those with IPA and 79.6% for those without an *Aspergillus* infection (*p* = 0.032). Assessment of clinical and laboratory features at IPA diagnosis is presented in Table 2. Of note, a trend pointing towards a longer median interval between ICU admission and IPA diagnosis in CAPA (6 days [IQR 3–13]) was observed compared to IAPA patients (3 days [IQR 2–5]; *p* = 0.14). Among patients treated by voriconazole, CAPA patients experienced a trend towards a longer time to reach therapeutic range (VTC target: 2–6 mg/L) (7 days [IQR 6–32] vs. 4 days [2,3,4,5,6,7,8], *p* = 0.096). Therapeutic drug monitoring of these patients showed a higher proportion of CAPA patients that obtained a delayed voriconazole therapeutic range since at day 5 after the initiation of voriconazole, 83.3% of CAPA patients and 33.3% of IAPA patients remained with a voriconazole dosage under 2 mg/L (*p* = 0.045). Similarly VTC appeared to be lower in CAPA patients (2.2 mg/L [IQR 1.1–4.4] vs. 3.9 mg/L [IQR 2–5.7]; *p* = 0.01).

**Figure 2:**
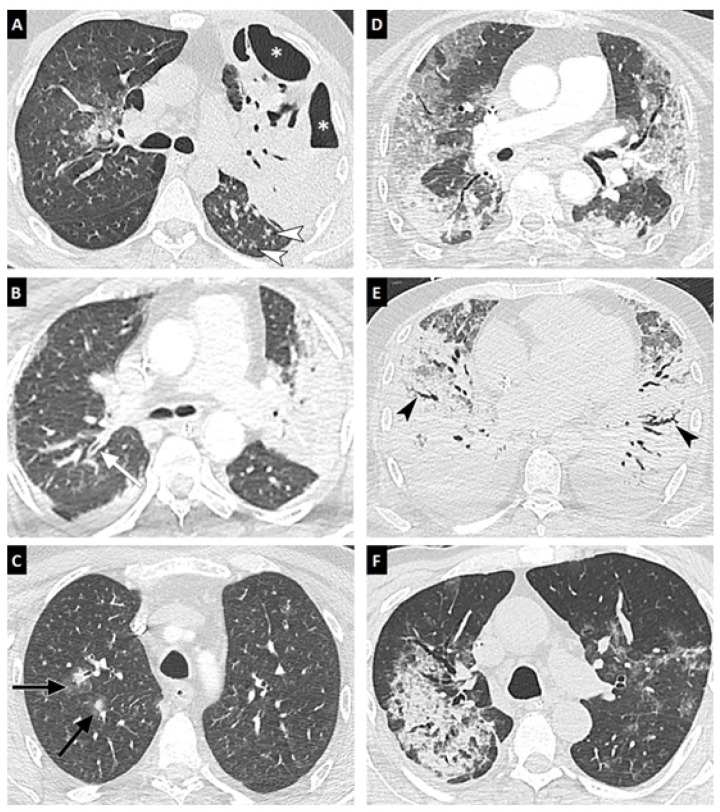
Chest CT-scan of LAPA and CAPA patients. Chest CT scans in the axial plane (lung window: W1600/L-500 HU) of three patients with LAPA (**A**–**C**) and three patients with CAPA (**D**–**F**). Typical CT findings in LAPA are unilateral or bilateral areas of consolidation with air bronchogram (**A**,**B**), cavity formation (asterisks), tree in bud (white arrowheads), bronchial wall thickening (white arrow), or occasionally nodules with halo signs (**C**, black arrows). Patients with CAPA may exhibit non-specific CT findings, such as bilateral areas of ground-glass opacity and/or crazy paving (**D**), extensive consolidations areas associated with peripheral traction bronchiectasis (**E**, black arrowheads), or, more rarely, unilateral consolidation areas (**F**). Despite not being very specific for a SARS COV2 infection, findings observed in (**D**,**E**) pictures can be seen frequently in severe COVID-19 patients.

Chest CT scans were performed for 24 of 27 patients between eight days before and 12 days after IPA diagnosis. Lung parenchyma abnormalities were present in all patients (Table 3). A lower proportion of well-circumscribed nodules, tree-in bud, and bronchial wall thickening was observed for CAPA than IAPA patients (0% vs. 42.9% [*p* = 0.024] for well-circumscribed nodules, 0% vs. 50% [*p* = 0.014] for tree in bud, and 10% vs. 57% [*p* = 0.03] for bronchial wall thickening). These different aspects are presented in Figure 2 and the comparison of CT-scan features of CAPA patients at admission and at diagnosis is presented in Table S3. 

Finally, a summary of similarities and differences between CAPA and IAPA are presented in Table 4.

## 4. Discussion

In this single-center study, 22.5% of patients admitted to the ICU for a severe viral infection, such as COVID-19 (20.4%) or Influenza pneumonia (23.9%), developed IPA. A similar prevalence of IPA in ICU patients was observed in the two major representative series of severe influenza (19.2% in a Dutch-Belgian multicenter study on 432 patients, [8]) and COVID-19 patients (19.6% probable and possible CAPA in a French multicenter prospective study with 509 patients included [16]). Secondary fungal infections may have had an impact on the prognosis for these patients, as the mortality rate, duration of mechanical ventilation, and ICU length of stay were associated with the occurrence of IPA. To date, only limited data are available on the comparison of IAPA and CAPA. In-hospital death occurred for 47.1% and 40% of patients with IAPA and CAPA, respectively, vs. 20.4% for patients without aspergillosis. Such a high mortality rate has already been observed for IAPA vs. non IAPA ICU patients (51% vs. 28%, respectively, [26]). Bartoletti et al., among others, have reported similar mortality rates of 44% for CAPA patients vs. 19% for non-CAPA patients during the COVID-19 pandemic [13]. Although the median age was significantly higher for CAPA than IAPA patients, other demographic data and characteristics of the patients at IPA diagnosis were comparable between the two populations. Among them, immunosuppression was not statistically significantly different, but 47.1% of IAPA patients were immunosuppressed vs. 20% of CAPA patients. Concerning biological data, lymphopenia, the PaO_2_ to FiO_2_ ratio on day 1, and the SOFA score on day 1 were less severe for the CAPA than IAPA patients.

IPA diagnosis in non-neutropenic ICU patients is challenging, as the clinical and radiological features of IPA are not specific and can be affected by underlying conditions. The radiological criteria of pulmonary mold diseases were recently revised in the consensus definitions for invasive fungal disease [1]. The authors proposed to add a new more sensitive item, defined as wedge-shaped lobar or segmental consolidation, to the classical CT criteria (solid nodule, halo sign, cavitation, air-crescent sign). These updated criteria were validated in a recent cohort analysis in which nodule and/or consolidation patterns were observed in more than 98% of IPA patients, irrespective of their neutrophil status [27]. Nodule and consolidation patterns were present, alone or together, in our study in 83% of cases (93% of IAPA and 70% of CAPA). Of note, although these updated criteria increase the sensitivity of CT for the detection of aspergillosis, they concurrently decrease its specificity, and there is currently no CT sign that is both sensitive and specific for aspergillosis. In the present study, although radiological findings were generally considered to not be suggestive of IPA during ARDS, a meticulous analysis showed several interesting differences between the images, with well-circumscribed nodules, tree in bud, and bronchial wall thickening, which were observed significantly more frequently in IAPA patients than CAPA patients. Otherwise, the timing of imaging may also affect the sensitivity of such tools regarding IPA diagnosis. It may be useful to perform new CT scans after the diagnosis of IPA in order to increase the sensitivity.

Because of these non-specific features, mycological testing is of great value for the screening of IPA in these patients. Compared to the AspICU classification, the main advance of the new consensual IAPA and CAPA definitions is to fine-tune criteria and tools for respiratory samples. In the IAPA definition according to Verweij et al., positive tracheal aspirates and even positive sputum cultures are criteria for probable IAPA, depending on the clinical presentation [28], and pulmonary or cavitating infiltrates [7]. Concerning CAPA, PCR and GM detection in respiratory samples are now included in the definition by Koehler et al. and although positive BAL is a criterion for probable CAPA, non-bronchoscopic respiratory samples are criteria for possible CAPA. The severity and clinical outcome of IAPA and CAPA from this series relative to that of other ICU patients highlights the relevance of these new definitions.

These specific conditions suggest that IPA is likely to be underdiagnosed in ARDS populations [4], whereas recent evidence has suggested that critically ill patients, in particular both severe influenza and COVID-19 patients, are populations at risk of IPA. Several mechanisms that may facilitate fungal infection have been identified, such as sepsis-induced systemic immunosuppression due to severe influenza virus or SARS-CoV-2 infection [29]. Several recent studies reported profound lymphopenia in severe COVID-19 associated with the expansion of myeloid derived suppressor cells, which may promote the acquisition of secondary infections, as illustrated by the large proportion of COVID-19 patients that develop respiratory reactivation of Herpes virus [30]. On the other hand, alveolar damage, dysfunction of mucociliary clearance, and local immune disorders due to COVID-19 or severe influenza pneumonia may also be key mechanisms involved in fungal invasion [8]. Finally, recent therapeutic strategies have emerged worldwide during the first months of COVID-19 pandemic with the aim of reducing inpatient mortality, such as corticosteroids, new antiviral drugs, or biotherapies. The recent RECOVERY trial [31] has positioned corticosteroids as first-line therapeutic agents, with a demonstrated improvement of patient prognosis. Although such improvement with low-dose corticosteroids (i.e., 6 mg of dexamethasone per day for 10 days) should be highlighted, we observed a significant association between corticosteroid use and the occurrence of IPA. Cohort studies have already demonstrated that corticosteroids increase the risk of IPA in severe influenza patients [11], emphasizing the need for enhanced awareness of IPA in these patients. Drug-drug interactions also arise in this context. Dexamethasone was widely used in CAPA patients and induces CYP2C9, which could decrease the VTC [32]. Thus, therapeutic drug monitoring is a cornerstone for patient management, as we faced low concentrations of voriconazole in this series with a significant delay to reach the optimal therapeutic range in CAPA vs. IAPA patients. Since we faced difficulties reaching voriconazole therapeutic concentrations in CAPA patients, the use of alternative antifungal treatments such as isavuconazole or posaconazole could be envisaged in this specific population [33,34].

Our study had several limitations, including the sample size, which prevented multivariate analysis. Furthermore, during the first wave of the COVID-19 pandemic, bronchoscopic explorations were considered as a source of exposure to SARS-CoV-2 for physicians due to the risk of the aerosolization during this procedure. Thus, we performed such investigations less frequently in COVID-19 patients; this risk has been debunked in recent papers [35]. Hence, it was not always possible to identify bronchoscopic findings suggestive of *Aspergillus* tracheobronchitis, such as epithelial plaques, pseudomembranes, or ulcers.

The main strengths of this study included the standardized management of ARDS and mycological testing of all patient samples, allowing an exhaustive laboratory data set. Furthermore, this is one of the first studies to apply the new consensual criteria for both IAPA and CAPA, whereas questions have been recently raised concerning the relevance of other classifications in determining the true burden of disease [6].

In conclusion, ICU patients presenting with ARDS during COVID-19 are very similar to those with severe influenza pneumonia in terms of the prevalence of IPA and outcome. It is now possible to draw the archetype of such patients using the new clinical and biological case definitions of IAPA and CAPA. Radiological findings of IPA in both populations using the new criteria increased the sensitivity but still lack specificity. Nevertheless, they also showed interesting differences between IAPA and CAPA. IAPA typically occurs earlier after ICU admission, in a more immunosuppressed and/or chronic respiratory disease background than CAPA, which is mainly favored by advanced age, irrespective of the medical background and with a lower rate of positivity of angioinvasion biomarkers. Finally, reaching voriconazole trough concentrations remains challenging in CAPA patients and emphasizes the importance of therapeutic drug monitoring. Future larger prospective studies may help in designing the most well-adapted personalized management to prevent IPA, which represents a high burden of death in severe COVID-19 and Influenza pneumonia.

## Figures and Tables

**Table 1:** Characteristics of patients according to aspergillosis status.

	All Patients (*n* = 120)	All Aspergillosis Patients (*n* = 27)	Non Aspergillosis Patients (*n* = 93)	*p* Value	IAPA (*n* = 17)	CAPA (*n* = 10)	*p* Value
Baseline characteristics							
Age (years)	59 (52–67)	60 (52–69)	59 (52–67)	0.54	58 (52–63)	72 (57–77)	0.036
Male sex	80 (66.4%)	17 (63%)	63 (67.7%)	0.21	11 (64.7%)	6 (60.0%)	>0.99
Current smoking	31 (26%)	10 (37%)	21 (22.6%)	0.13	9 (52.9%)	1 (10.0%)	0.12
Obesity	20 (16.6%)	4 (15.4%)	30 (32.3%)	0.09	3 (17.6%)	1 (10.0%)	>0.99
Diabetes	33 (27.5%)	4 (14.8%)	29 (31.2%)	0.14	2 (11.8%)	2 (20.0%)	0.61
Alcoholism	20 (16.6%)	6 (22.2%)	14 (15.1%)	0.39	5 (29.4%)	1 (10.0%)	0.36
Immunodepression (including neoplasia)	29 (24.1%)	10 (37%)	19 (20.4%)	0.12	8 (47.1%)	2 (20.0%)	0.23
Neoplasia	19 (15.8%)	7 (25.9%)	12 (12.9%)	0.13	6 (35.3%)	1 (10.0%)	0.2
- Solid cancer	3 (2.5%)	1 (3.7%)	2 (2.2%)	0.53	1 (5.9%)	0 (0.0%)	>0.99
- HM	16 (13.3%)	6 (22.2%)	10 (10.7%)	0.19	5 (29.4%)	1 (10.0%)	0.36
Chronic obstructive pulmonary disease	17 (14.2%)	6 (22.2%)	11 (11.8%)	0.17	6 (35.3%)	0 (0.0%)	0.057
Chronic kidney disease	10 (8.3%)	2 (7.4%)	8 (8.6%)	>0.99	1 (5.9%)	1 (10.0%)	>0.99
Cirrhosis	8 (6.7%)	4 (14.8%)	4 (4.3%)	0.07	4 (23.5%)	0 (0.0%)	0.26
ARDS etiology					NA	NA	NA
- Influenza	71 (59.2%)	17 (63%)	54 (58.1%)	0.65			
- COVID-19	49 (40.8%)	10 (37%)	39 (41.9%)				
Clinical and biological admission ICU data							
Neutrophil (10^9^/L)	6.9 (3.9–11.4)	8.2 (3.8–13.2)	6.9 (4.1–11)	0.67	8.0 (3.6–17.7)	8.5 (4.1–11.0)	0.72
Lymphocyte (10^9^/L)	0.56 (0.32–0.87)	0.54 (0.36–0.72)	0.59 (0.32–0.93)	0.44	0.38 (0.29–0.55)	0.83 (0.72–0.92)	<0.0001
Ratio of PaO2 to FiO2 on day 1	98 (67–147)	98 (74–143)	105 (67–148)	0.96	86 (69–98)	143 (109–154)	0.01
SAPS II score on day 1	44 (35–61)	48 (36–64)	43 (34–60)	0.22	58 (42–64)	40 (34–68)	0.48
SOFA score on day 1	8 (5–10)	9 (5–12)	7 (4–10)	0.19	10 (7–13)	5 (2–8)	0.012
Clinical course data							
Duration of mechanical ventilation (days)	18 (11–27)	23 (17–40)	17 (9–25)	0.038	23 (16–49)	23 (19–30)	0.56
ECMO	45 (37.5%)	13 (48.1%)	32 (34.4%)	0.19	12 (70.6%)	1 (10.0%)	0.004
SOFA score on day 5	8 (6–12)	11 (7–14)	7 (5–11)	0.003	10 (6–14)	12 (8–13)	0.83
RRT use	37 (30.8%)	13 (48.1%)	24 (25.8%)	0.027	8 (47.1%)	5 (50.0%)	>0.99
Corticosteroids use	55 (45.8%)	19 (70.4%)	36 (38.7%)	0.004	12 (70.6%)	7 (70.0%)	>0.99
- before day 7	45 (81.8%)	16 (84.2%)	29 (80.6%)	0.008	12 (100%)	4 (57.1%)	0.22
- after day 7	10 (18.2%)	3 (15.8%)	7 (19.4%)	0.14	0 (0.0%)	3 (42.9%)	0.04
ICU length of stay (days)	22 (12–33)	25 (19–48)	19 (12–30)	0.044	29 (12–48)	24 (22–29)	0.97
Death in the ICU	28 (23.3%)	9 (33.3%)	19 (20.4%)	0.16	6 (35.3%)	3 (30.0%)	>0.99
90-day survival	89 (74.2%)	16 (59.3%)	74 (79.6%)	0.032	9 (52.9%)	7 (70.0%)	0.44
Death in hospital	31 (25.8%)	12 (44.4%)	19 (20.4%)	0.012	8 (47.1%)	4 (40.0%)	0.45

Data are presented as medians (IQR: interquartiles) or *n* (%). *p* values comparing the invasive aspergillosis vs. no aspergillosis groups and IAPA vs. CAPA were calculated using Mann–Whitney (continuous variables) and Fisher or Chi2 tests when appropriate (categorical variables). AKI: acute kidney injury, PaO2: arterial oxygen partial pressure, SAPS II: Simplified Acute Physiology Score I, SOFA: Sequential Organ Failure Assessment, HM: hematological malignancies, COVID-19: Coronavirus Disease 2019, ECMO: extracorporeal membrane oxygenation; PaO2: arterial oxygen tension; FiO2: fraction of inspired oxygen; RRT: renal replacement therapy; ICU: intensive care unit; - among neoplasia.

**Table 2:** Clinical and biological characteristics at CAPA and IAPA diagnosis.

	All Aspergillosis Patients (*n* = 27)	IAPA (*n* = 17)	CAPA (*n* = 10)	*p* Value
Temperature (°C)	38.2 (38.0–39.0)	38.0 (37.8–38.8)	38.9 (38.1–39.0)	0.19
Systolic pressure (mmHg)	92 (81–102)	92 (85–102)	90 (78–101)	0.95
Neutrophil count (10^9^/L)	9.6 (4.5–16.5)	13.2 (5.5–19.5)	7.6 (4.0–10.3)	0.18
Lymphocyte count (10^9^/L)	0.72 (0.51–1.03)	0.80 (0.50–1.22)	0.72 (0.55–0.80)	0.56
Ratio of PaO2 to FiO2	134 (102–179)	108 (86–165)	162 (147–208)	0.04
Septic shock	17 (63.0%)	12 (70.6%)	5 (50.0%)	0.41
Need for vasopressors	19 (70.4%)	12 (70.6%)	7 (70.0%)	>0.99
Delay between admission and aspergillosis onset (days)	4 (2–8)	3 (2–5)	6 (3–13)	0.14
Mechanical ventilation duration after aspergillosis onset (days)	20 (9–36)	22 (8–46)	17 (11–23)	0.64
Antifungal therapy	25 (92.6%)	17 (100%)	8 (80%)	0.13
Time to VCZ therapeutic range (days) *	6 (4–9)	4 (2–8)	7 (6–32)	0.096
Delayed VCZ therapeutic range (>5 days) *	9/18 (50%)	4/12 (33.3%)	5/6 (83.3%)	0.045
VTC (mg/L) *	2.8 (1.5–5)	3.9 (2–5.7)	2.2 (1.1–4.4)	0.01
VTC min (mg/L) *	1.6 (0.5–3.8)	3.5 (1–5)	0.8 (0.2–0.8)	0.038
VTC max (mg/L) *	5.8 (4.5–7.2)	6.1 (5.4–7.6)	5.2 (3–7)	0.23
Antifungal treatment duration (days)	42 (14–42)	17 (14–47)	42 (37–42)	0.49

Data are presented as medians (IQR: interquartiles) or *n* (%). *p* values comparing IAPA vs. CAPA were calculated using Mann–Whitney (continuous variables) and Fisher or Chi2 tests when appropriate (categorical variables). IAPA: Influenza Associated Pulmonary Aspergillosis; CAPA: COVID-19 Associated Pulmonary Aspergillosis; PaO2: arterial oxygen partial pressure; FiO2: Fraction of inspired Oxygen; VCZ: Voriconazole; VTC: Voriconazole Trough Concentration. * Among IPA patients, 19 were treated by voriconazole alone and 18 of them were performed a watchful therapeutic drug monitoring, two patients were treated by isavuconazole, two others by voriconazole which was switched for isavuconazole, one by voriconazole plus amphotericin B, and finally two patients did not receive any antifungal treatment. The data concerning TDM monitoring displayed in this table are based on the results of the 18 patients who only received voriconazole and for whom frequent monitoring could be performed.

**Table 3:** CT-scan analysis of IAPA and CAPA patients.

	All Aspergillosis Patients (*n* = 24)	IAPA (*n* = 14)	CAPA (*n* = 10)	*p* Value
Delay between ICU admission and CT scan	10 (4–15)	9 (4–15)	10 (3–15)	0.99
Delay between IPA diagnosis and CT scan	5 (0–9)	4 (0–9)	7 (0–9)	0.75
Diffuse reticular or alveolar opacities	24 (100%)	14 (100%)	10 (100%)	0.99
Wedge-shaped segmental or lobar consolidation	17 (70.8%)	10 (71.4%)	7 (70.0%)	0.99
Well-circumscribed nodule(s)	6 (25.0%)	6 (42.9%)	0 (0.0%)	0.024
Halo sign	3 (12.5%)	2 (14.3%)	1 (10.0%)	0.68
Cavitation	5 (20.8%)	5 (35.7%)	0 (0.0%)	0.053
Air-crescent sign	0 (0.0%)	0 (0.0%)	0 (0.0%)	0.99
Tree in bud	7 (29.2%)	7 (50.0%)	0 (0.0%)	0.019
Bronchial wall thickening	8 (33.3%)	8 (57.1%)	1 (10.0%)	0.03
Pleural effusion	9 (37.5%)	5 (35.7%)	4 (40.0%)	0.99

Data are presented as *n* (%). *p* values comparing influenza associated pulmonary aspergillosis (IAPA) vs. COVID-19 associated pulmonary aspergillosis (CAPA). IAPA and CAPA groups were tested using Fisher’s exact test (categorical variables). CT-scan: computerized-tomography scanner.

**Table 4:** Summary of similarities and differences between CAPA and IAPA.

Similarities between CAPA and IAPA	Differences between CAPA and IAPA
Prevalence of IPA between COVID-19 and influenza-associated ARDS Background of patients Similar clinical courses in ICU with a trend for a longer median interval between ICU admission and IPA diagnosis in CAPA Higher mortality than for patients without IPA among both CAPA and IAPA patients	Higher proportion of older patients among CAPA patients Lower day 1 SOFA score in CAPA patients Higher ratio of PaO2 to FiO2 in CAPA patients Lower proportion of ECMO among CAPA patients Therapeutic drug monitoring of voriconazole more challenging for CAPA patients Lower proportion of patients presenting radiological features suggestive of IPA among CAPA patients

## Data Availability

Data are availbale in the Clinical data center of our hospital.

## Supplementary Materials

The following are available online at https://www.mdpi.com/article/10.3390/jof7050388/s1, Table S1: Mycological arguments for invasive pulmonary aspergillosis associated to influenza pneumonia (IAPA) and COVID-19 (CAPA) and classification, Table S2: Summary of reported cases of influenza associated pulmonary aspergillosis (IAPA) and COVID-19 associated pulmonary aspergillosis (CAPA). Table S3: Comparison of CT-scan features of CAPA petients at admission and at diagnosis.
