# Supplementary material for: Influenza- and COVID-19-Associated Pulmonary Aspergillosis: Are the Pictures Different?
Source: J Fungi (Basel). 2021 May 15;7(5):388. doi: 10.3390/jof7050388 (PMC8156373; doi:10.3390/jof7050388)
Supplement: Supplementary file 1 [file jof-07-00388-s001.zip › jof-1202848-supplementary.pdf]

Table S1. Mycological arguments for invasive pulmonary aspergillosis associated to influenza pneumonia (IAPA) and COVID-19 (CAPA) and classification.

Patient	Age/Sex	Positive Respiratory Samples	Blood Samples	IAPA Classification	CAPA Classification
#1	57/F	Positive culture with *A. fumigatus*: 1 BAL and 1 TBA<br>Positive BAL for: 1 x GM≥1 ; 1 × *A. fumigatus* PCR	1 x Positive GM>0.5	Probable IAPA*	
#2	40/M	Positive culture with *A. fumigatus*: 1 TBA<br>Positive TBA for: 1 x *A. fumigatus* PCR	Negative (x 3)	Putative IPA**	
#3	63/M	Positive culture with *A. fumigatus*: 1 BAL and 1 TBA<br>Positive BAL for: 2 x GM≥1 ; 1 x *A. fumigatus* PCR	1 x Positive GM>0.5	Probable IAPA	
#4	54/F	Positive culture with *A. fumigatus*: 1 BAL and 1 TBA<br>Positive BAL for: 1 x GM≥1 ; 1 x *A. fumigatus* PCR	Negative (x 1)	Probable IAPA	
#5	69/M	Positive BAL for: 1 x GM≥1<br>Positive TBA for: 1 x GM≥1	Negative (x 3)	Probable IAPA	
#6	58/M	Positive culture with *A. fumigatus*: 1 BAL<br>Positive BAL for: 1 x GM≥1 ; 1 x *A. fumigatus* PCR	2 x Positive GM>0.5	Probable IAPA	
#7	67/F	Positive BAL for: 1 x GM≥1	2 x Positive GM>0.5	Probable IAPA	
#8	49/M	Positive culture with *A. fumigatus*: 1 TBA<br>Positive TBA for: 1 x GM≥1	Negative (x 4)	Putative IPA	
#9	34/F	Positive culture with *A. fumigatus*: 1 BAL<br>Positive BAL for: 1 x GM≥1 ; 1 x *A. fumigatus* PCR	Negative (x 2)	Probable IAPA	
#10	60/M	Positive culture with *A. fumigatus*: 1 TBA<br>Positive TBA for: 2 x GM≥1 ; 2 x *A. fumigatus* PCR	Negative (x 5)	Putative IPA	
#11	67/M	Hyphae consistent with *Aspergillus* in a TBA<br>Positive culture with *A. fumigatus*: 1 TBA<br>Positive TBA for: 1 x *A. fumigatus* PCR	Negative (x 1)	Probable IAPA	
#12	51/M	Positive culture with *A. fumigatus*: 1 BAL<br>Positive BAL for: 1 x *A. fumigatus* PCR	Negative (x 1)	Probable IAPA	
#13	52/M	Positive culture with *A. fumigatus*: 1 BAL<br>Positive BAL for: 2 x GM≥1; 1 x *A. fumigatus* PCR	1 x Positive GM>0.5	Probable IAPA	
#14	63/F	Hyphae consistent with *Aspergillus* in 1 TBA<br>Positive culture with *A. fumigatus*: 1 TBA<br>Positive TBA for: 1 x GM≥1	1 x Positive GM>0.5	Probable IAPA	
#15	57/F	Positive culture with *A. fumigatus*: 1 BAL, 1 TBA and 1 sputum<br>Positive BAL for: 1 x GM≥1; 1 x *A. fumigatus* PCR<br>Positive TBA and sputum for: 1 x *A. fumigatus* PCR	2 x Positive GM>0.5	Probable IAPA	

#16	62/M	Hyphae consistent with *Aspergillus* in a 1 BAL, 1 TBA and 1 sputum<br>Positive culture with *A. fumigatus*: 1 TBA and 1 sputum<br>Positive BAL for: 1 x GM≥1<br>Positive TBA and sputum for: 1x GM≥1; 1 x *A. fumigatus* PCR	Negative (x 1)	Probable IAPA	
#17	60/M	Positive culture with *A. fumigatus*: 2 TBA<br>Positive TBA for: 1 x GM≥1	Negative (x 3)	Putative IPA	
#18	48/F	Positive culture with *A. fumigatus*: 1 TBA<br>Positive TBA for: 5 x *A. fumigatus* PCR	Negative (x 1)		Possible CAPA
#19	78/F	Positive culture with *A. fumigatus*: 2 TBA<br>Positive TBA for: 2 x *A. fumigatus* PCR	1 x Positive GM>0.5		Probable CAPA
#20	78/F	Positive culture with *A. fumigatus*: 4 TBA<br>Positive TBA for: 4 x GM≥1.2 (2 x >4.5); 6 x *A. fumigatus* PCR	Negative (x 2)		Possible CAPA
#21	71/M	Positive culture with *A. fumigatus*: 1 TBA<br>Positive TBA for: 1 x *A. fumigatus* PCR	Negative (x 2)		Possible CAPA
#22	73/M	Positive culture with *A. fumigatus*: 2 TBA<br>Positive TBA for: 4 x GM≥1.2 ; 5 x *A. fumigatus* PCR	Negative (x 1)		Possible CAPA
#23	75/M	Positive culture with *A. fumigatus*: 4 TBA<br>Positive TBA for: 2 x *A. fumigatus* PCR	Negative (x 2)		Possible CAPA
#24	53/M	Positive TBA for: 1 x GM≥1.2; 1 x *A. fumigatus* PCR	Negative (x 1)		Possible CAPA
#25	46/F	Positive culture with *A. fumigatus*: 1 sputum	1 x Positive GM>0.5		Probable CAPA
#26	82/F	Hyphae consistent with *Aspergillus* in a 1 BAL<br>Positive culture with *A. fumigatus*: 1 TBA<br>Positive TBA for: 3 x *A. fumigatus* PCR	Negative (x 1)		Probable CAPA
#27	69/M	Positive culture with *A. fumigatus*: 5 TBA<br>Positive TBA for: 5 x GM≥1.2 (5 x >4.5); 7 x *A. fumigatus* PCR	2 x Positive GM>0.5		Probable CAPA

GM: galactomannan. BAL: broncho-alveolar lavage ; TBA : tracheo-bronchial aspiration.* Probable IAPA: According to Verweij et al. (7). **Putative IPA: according to Blot et al. (3)

Table S2. Summary of reported cases of influenza associated pulmonary aspergillosis (IAPA) and COVID-19 associated pulmonary aspergillosis (CAPA).

Case number	Age/ Sex	SAPS-II at ICU Admission	Corticosteroids before IA	Aspergillosis Risk Factors	Delay before IPA, Days	Aspergillosis Classification	Radiological Evidence	Antifungal Therapy	Outcome (day 90)
1	57/F	60	Hydrocortisone, 13d	Chemotherapy, breast cancer	7	Probable IAPA*	Diffuse reticular or alveolar opacities well-shaped nodules	Voriconazole, 3d	dead
2	40/M	22	Hydrocortisone, 5d	Severe cirrhosis	10	Putative IPA**	Diffuse reticular or alveolar opacities, pleural fluid, wedge-shaped infiltrate cavitation and tree in bud	Voriconazole, 17d	alive
3	63/M	82	Hydrocortisone, 5d	n	4	Probable IAPA	Diffuse reticular or alveolar opacities, Wedge-shaped infiltrate, cavitation and tree in bud	Voriconazole + isavuconazole, 117d	alive
4	54/F	86	n	n	3	Probable IAPA	Diffuse reticular or alveolar opacities and wedge-shaped infiltrate	Voriconazole, 60d	alive
5	69/M	65	n	Hodgkin Lymphoma	5	Probable IAPA	Diffuse reticular or alveolar opacities well-shaped nodules and cavitation	Voriconazole, 14d	dead
6	58/M	47	Hydrocortisone, 3d	n	3	Probable IAPA	Diffuse reticular or alveolar opacities and wedge-shaped infiltrate	Voriconazole, 2d	dead
7	67/F	64	n	Myeloma	3	Probable IAPA	Diffuse reticular or alveolar opacities, wedge-shaped infiltrate, pleural fluid, well-shaped nodules, halo sign and tree in bud	Voriconazole, 9d	dead
8	49/M	61	Hydrocortisone, 9d	Chronic lymphocytic leukemia	4	Putative IAPA	Diffuse reticular or alveolar opacities, nonspecific infiltrates and consolidation	Voriconazole, 54d	alive
9	34/F	47	Methylprednisolone, 20d	n	2	Probable IAPA	Diffuse reticular or alveolar opacities, wedge-shaped infiltrate, pleural fluid, well-shaped nodules and tree in bud	Voriconazole, 42d	alive

10	60/M	37	Hydrocortisone, 7d	n	2	Putative IPA	Diffuse reticular or alveolar opacities, nonspecific infiltrates and consolidation, pleural fluid and wedge-shaped infiltrate	Voriconazole, 47d	alive
11	67/M	48	Hydrocortisone, 8d	Corticosteroids + TNF alpha inhibitors	1	Probable IAPA	Diffuse reticular or alveolar opacities and tree in bud	Voriconazole, 14d	dead
12	51/M	58	Hydrocortisone + methylprednisolone, 5d	Hodgkin lymphoma	0	Probable IAPA	Diffuse reticular or alveolar opacities, well-shaped nodules, halo sign and tree in bud	Voriconazole, 42d	alive
13	52/M	68	Methylprednisolone, 8d	n	0	Probable IAPA	Diffuse reticular or alveolar opacities, wedge-shaped infiltrate, well-shaped nodules and cavitation	Voriconazole, 17d	alive
14	63/F	59	n	n	2	Probable IAPA	Diffuse reticular or alveolar opacities, pleural fluid, wedge-shaped infiltrate and cavitation	Voriconazole, 5d	dead
15	57/F	32	Hydrocortisone, 20d	Myeloma	2	Probable IPA	Diffuse reticular or alveolar opacities, nonspecific infiltrates and consolidation and wedge-shaped infiltrate	Voriconazole, 16d	dead
16	62/M	29	n	n	9	Probable IAPA	Diffuse reticular or alveolar opacities, wedge-shaped infiltrate and cavitation	Voriconazole + amphotericin B, 42d	alive
17	60/M	42	n	n	17	Putative IPA	Diffuse reticular or alveolar opacities, nonspecific infiltrates and consolidation, pleural fluid and wedge-shaped infiltrate	Voriconazole, 32d	dead
18	48/F	86	Methylprednisolone, 20d	Lung transplantation	6	Possible CAPA	Nonspecific infiltrates and consolidation and Wedge-shaped infiltrate	Isavuconazole, 42d	alive

19	78/F	81	Hydrocortisone, 7d	Chronic myelo-mono-cytic leukemia	13	Probable CAPA	Diffuse reticular or alveolar opacities and nonspecific infiltrates and consolidation	Voriconazole, 2d	dead
20	78/F	37	n	n	4	Possible CAPA	Diffuse reticular or alveolar opacities and Wedge-shaped infiltrate	Voriconazole, 21d	dead
21	71/M	35	n	n	13	Possible CAPA	Diffuse reticular or alveolar opacities, nonspecific infiltrates, consolidation, pleural fluid and wedge-shaped infiltrate	Voriconazole, 42d	alive
22	73/M	34	Methylprednisolone, 7d	n	21	Possible CAPA	Diffuse reticular or alveolar opacities and pleural fluid	Isavuconazole, 42d	alive
23	75/M	42	Methylprednisolone, 30d	n	5	Possible CAPA	Diffuse reticular or alveolar opacities and Wedge-shaped infiltrate	Voriconazole, 42d	alive
24	53/M	17	n	n	3	Possible CAPA	Diffuse reticular or alveolar opacities and wedge-shaped infiltrate	none	alive
25	46/F	57	Methylprednisolone, 10d	n	28	Probable CAPA	Nonspecific infiltrates and consolidation and wedge-shaped infiltrate	none	alive
26	82/F	72	Hydrocortisone, 10d	n	1	Probable CAPA	Diffuse reticular or alveolar opacities and Wedge-shaped infiltrate and pleural fluid	Voriconazole + isavuconazole, 42d	dead
27	69/M	30	n	n	1	Probable CAPA	Diffuse reticular or alveolar opacities and Nonspecific infiltrates and consolidation	Voriconazole + isavuconazole, 42d	alive

* Probable IAPA: According to Verweij et al. (7). **Putative IPA: according to Blot et al. (3).

Table S3. Comparison of CT-scan features of CAPA patients at admission and at diagnosis.

	CT Scan at Admission ($n = 9$)	CT Scan at CAPA Diagnosis ($n = 10$)	P Value
Typical COVID-19 pattern	7 (78%)	3 (30%)	0.07
Diffuse reticular or alveolar opacities	7 (78%)	10 (100%)	0.21
Wedge-shaped segmental or lobar consolidation	6 (66.7%)	7 (70.0%)	0.86
Well-circumscribed nodule(s)	0 (0.0%)	0 (0.0%)	0.99
Halo sign	0 (0.0%)	1 (10.0%)	0.99
Cavitation	0 (0.0%)	0 (0.0%)	0.99
Air-crescent sign	0 (0.0%)	0 (0.0%)	0.99
Tree in bud	0 (0.0%)	0 (0.0%)	0.99
Bronchial wall thickening	1 (11.1%)	1 (10.0%)	0.99
Pleural effusion	1 (11.1%)	4 (40.0%)	0.3

Data are presented as n (%). P values comparing CT-scan analysis of CAPA patients at admission versus CT-scan of these patients at IPA diagnosis. CT-scan: computerized-tomography scanner .
